# Hints for the Sick Room

**Published:** 1889-03-30

**Authors:** M. M. Basil

**Affiliations:** Author of "The Commoner Accidents to Life and Limb"


### Hints for the Sick-room.

By M. M. Basil, M.A., M.B., C.M., Author of "The Commoner Accidents to Life and Limb."

*(Concluded from p. 398.)*

### THE IMPORTANCE OF POSTURE.

Towards the termination of serious diseases the question of posture becomes a matter of life and death. Many prolonged diseases, not affecting the chest in the first instance, really kill the patient by interfering with the action of the lungs. The patient dies at the lungs, though not suffering from lung disease. The lower and posterior portions of the lungs become choked up, so to speak, by blood collecting there by gravitation during the weak state of the entire system. Indeed, so frequent is this additional phenomenon in weakening disease that it is known, among other names, as the engorgement of position, or the pneumonia of the dying. Lying flat on the back, like a log of wood, adds to the choking up of the lower portions of the lungs. The commencement of it is betrayed by increased number of respirations, by difficulty felt by the patient in breathing, and by movement of the wings of the nose at each respiration. The last symptom is a most important warning to the nurse. The patient must be supported into the semi-sitting position at once, and turpentine stupes or stimulating poultices applied round the chest. A similar state of matters occurs, especially in old people, in the cavity of the brain, and the head must therefore be supported up, or its position frequently changed from side to side. This point must be attended to in all cases of coma.

Strict attention to position may be of vital importance in the nursing of a patient. There are many cases placed on record where even slight alterations in this respect have led to fatal results. One case is alluded to by Sir Thomas Watson, where, in disease of the cervical vertebræ, a slight change killed the patient at once. In another case, mentioned by the same writer, the barber gave a fatal twist while shaving the head for the application of cold locally. In two other recorded cases of cervical disease fatal results followed changes of position, effected by the nurse against distinct instructions given to her, but very likely without an explanation of the reasons for such caution. Sudden changes of position—and the same remark is true of all forms of excitement—may lead to instantaneous death in rheumatic fever and acute heart diseases, in aneurism, in diphtheria, scurvy, purpura, beriberi, and dengue. The last two in this list are tropical diseases, but, nevertheless, extremely important to us. No disease which endangers the lives of thousands of the Queen's subjects can be anything but of painful interest to the English nurse. The erect position may lead to serious results in typhoid fever and severe diarrhœa or dysentery.

In cases of vomiting, accompanied with pain over the stomach, lying on the left side will often relieve the vomiting which has resisted many forms of treatment.

Even a slight alteration in the position of the patient may be an important symptom of improvement. In low fevers, for example, turning on to the side after lying on the back for days is a symptom which you will learn by experience to hail with joy.

You must attend strictly to position after operations of even very slight nature, such as the introduction of an exploratory needle into the chest or a tumour. Should the patient die after sitting up suddenly, or from other errors of nursing, the friends are apt to overlook the immediate cause of death really at work, and remember the needle and the "operation" for many years.

We shall see later on that elevating the seat of hæmorrhage is one of the readiest and best means of checking bleeding. It would be well to remember that this principle must not be applied in cases of abdominal or pelvic bleeding. The blood will do less harm if it is allowed to collect in the pelvis, compared with its results when brought in contact with the organs situated higher up in the abdomen.

In the performance of the duties under consideration it is of great assistance to proceed systematically. A chart or programme of the present, past, and future events of the case is a valuable guide. On it should be marked the temperature, pulse, respiration, number of stools, diet to be taken, diet actually taken, and other features of the case as they present themselves. You must observe the facts rigidly as to time and circumstance, and note them down on the spot. The quantity of liquids taken, secreted or discharged, must be measured, not merely expressed by vague terms. The report which comes from you to the doctor must be strictly divided into two classes; first, facts which have been observed by yourself, and secondly, reports made to you by the patient and others. None of this second group must be represented to the medical attendant as facts personally observed. In ascertaining clinical facts you must believe no man or woman, otherwise there will be weeping and gnashing of teeth. Reports coming from the patient are looked upon differently from facts observed by a trained nurse. Every one is on the look-out to avoid stumbling over the first group; few think the same caution and healthy scepticism called for in dealing with the second group. Hence the irritation is all the greater after stumbling in a place where smooth walking was expected as a right.

The reports made by a patient may be expressed in the words used by her. Here you undertake no responsibility for the statement made. But should you undertake to communicate the thoughts of the patient, and not merely her words, it is necessary to make out exactly what she means by her terms. A patient often complains of pain in the heart when she means pain low down in the pelvis, or of pain in her kidneys when she means pain high up between the shoulders; or blames the leg when the hypogastrium is meant: but these expressions, if appropriated by the nurse, and reported as her own ideas, may cause serious error. Then, again, you must be sure that the patient understands your questions in the sense you intend them to be taken. Some patients make distinctions too profound for ordinary comprehension. Not unfrequently a patient, after saying that she has had no vomiting, adds the rider, "but nothing she takes will stay on her stomach." Finally, the Celt repeatedly asserts that a limb is "very painful, but he does not feel the pain." Probably this is his peculiar interpretation of abnormal sensations, such as pins-and-needles, ant-creeping, and other conditions.

### FELLOWSHIP.

On Hospital Sunday, at Romford, the Rev. F. E. Allen preached to many members of the various friendly societies of the town. He took as his text: "Whether one member suffers, all the rest suffer with it," 1 Cor. xii., 26. He said that men, unlike the lower creation, were naturally inclined to band themselves together in families, in tribes, and in nations, and it was that principle which led members of friendly societies to associate themselves together for mutual benefit. Those who had founded societies such as their own, had laid hold of the principle enunciated by the Apostle—the principle that we were not mere units. The preacher then dwelt at some length upon the spiritual aspect of the text. Having compared the unity which existed between the different members of the body with the unity which existed between Christ and His Church, he said that there might probably be some present who did not think the Church had been so anxious to benefit the working classes as she ought to have been. Whilst he was free to confess that she had not always been so attractive or loving towards them as she ought to have been, he was very sure the fault had been with those who were members of the Church, and not the fault of the constitution of the Church itself. And he was also very sure that she was now waking up more as the true mother of all her sons in England than perhaps she had ever been in the past.

